# Attitudes and beliefs towards transgender individuals among residents of Mashhad in 2020

**DOI:** 10.1192/j.eurpsy.2022.719

**Published:** 2022-09-01

**Authors:** S. Omidvar Tehrani, A. Talaei, B. Sorouri Khorashad, F. Afzaljavan

**Affiliations:** 1 Mashhad University of Medical Sciences, Psychiatry And Behavioral Sciences Research Center, Mashhad, Iran; 2 Karolinska Institute, Department Of Women’s And Children’s Health, Stockholm, Sweden

**Keywords:** Transgender, transphobia, attitude, beliefs

## Abstract

**Introduction:**

Transgender people are more vulnerable to psychiatric morbidities compared to cisgender people. This increased vulnerability can be partly due to the discrimination and stigma transgender people experience.

**Objectives:**

Several studies have tried to assess the stigma by studying the public attitudes and beliefs about transgender people. This study aims to explore the attitudes of a large sample of Iranian citizens toward transgender people.

**Methods:**

In this cross-sectional study, attitudes and beliefs towards transgender individuals were evaluated using the GTS among citizens of the Mashhad city of Iran. Participants were interviewed, and demographic data and socio-economic status of participants were also obtained.

**Results:**

A total of 1202 participants with a mean age of 41.57±13.41, including 27.4% males and 72.6% females, participated in the study. In our sample, the GTS mean score indicated a moderately positive attitude toward transgender individuals. Our results pointed out the significant difference between sex (p=0.002), marital status (p<0.001), educational and economic levels (p<0.001) in GTS. Furthermore, people who knew a Transgender individual indicated higher GTS (p<0.001).

**Conclusions:**

In Iran, with religious culture and a closed community, the situation for transgender people can be more challenging compared to that of Western countries. Although intolerant views toward transgender people have faded in recent years, society’s attitude is still negative. This investigation revealed that educational level accounted for much of the variance in transgender attitudes. Therefore, we can say that increasing informative trans-related content in social media can educate the general population and reduce anti-trans attitudes and behaviors.

**Disclosure:**

No significant relationships.

